# Histone H3K56 Acetylation, CAF1, and Rtt106 Coordinate Nucleosome Assembly and Stability of Advancing Replication Forks

**DOI:** 10.1371/journal.pgen.1002376

**Published:** 2011-11-10

**Authors:** Marta Clemente-Ruiz, Román González-Prieto, Félix Prado

**Affiliations:** Departamento de Biología Molecular, Centro Andaluz de Biología Molecular y Medicina Regenerativa (CABIMER), Consejo Superior de Investigaciones Científicas (CSIC), Seville, Spain; Brandeis University, United States of America

## Abstract

Chromatin assembly mutants accumulate recombinogenic DNA damage and are sensitive to genotoxic agents. Here we have analyzed why impairment of the H3K56 acetylation-dependent CAF1 and Rtt106 chromatin assembly pathways, which have redundant roles in H3/H4 deposition during DNA replication, leads to genetic instability. We show that the absence of H3K56 acetylation or the simultaneous knock out of CAF1 and Rtt106 increases homologous recombination by affecting the integrity of advancing replication forks, while they have a minor effect on stalled replication fork stability in response to the replication inhibitor hydroxyurea. This defect in replication fork integrity is not due to defective checkpoints. In contrast, H3K56 acetylation protects against replicative DNA damaging agents by DNA repair/tolerance mechanisms that do not require CAF1/Rtt106 and are likely subsequent to the process of replication-coupled nucleosome deposition. We propose that the tight connection between DNA synthesis and histone deposition during DNA replication mediated by H3K56ac/CAF1/Rtt106 provides a mechanism for the stabilization of advancing replication forks and the maintenance of genome integrity, while H3K56 acetylation has an additional, CAF1/Rtt106-independent function in the response to replicative DNA damage.

## Author Summary

Loss of replication fork integrity is a primary source of genetic instability. In eukaryotes DNA synthesis is rapidly followed by its assembly into chromatin, and these two processes are tightly connected. Defective chromatin assembly mutants accumulate DNA damage and are sensitive to genotoxic agents, even though the mechanisms responsible for this genetic instability remain unclear because chromatin assembly also plays essential roles in transcription, silencing, DNA repair, and checkpoint signaling. A good example is the acetylation of histone H3 at lysine 56, which promotes histone deposition by the chromatin assembly factors CAF1 and Rtt106. In this case, the absence of this modification also causes a loss of structural and/or coding information at chromatin. Here we show that defective replication-coupled chromatin assembly leads to an accumulation of recombinogenic DNA damage by affecting the integrity of advancing, but not stalled, replication forks. Therefore, we propose that H3K56ac/CAF1/Rtt106-dependent chromatin assembly provides a mechanism for the stabilization of replication forks. Besides, H3K56 acetylation promotes replicative DNA damage repair/tolerance through a function that is independent of CAF1/Rtt106 and likely subsequent to its deposition at chromatin, revealing this modification as a key regulator of genome integrity.

## Introduction

Problems in DNA replication are a direct cause of genetic instability and are associated with early tumor development [1]. This instability is linked to a high susceptibility of the replication forks to become stalled, damaged or even broken, and for this reason understanding of the scenarios that threaten replication fork integrity is crucial, but also the mechanisms that promote replication fork repair and restart. Cells are endowed with a complex network of checkpoints mechanisms that coordinate DNA damage repair with cell cycle progression [2]. Thus, during S phase, arrested or damaged forks trigger a signal transduction cascade ending up in the phosphorylation of effector kinases (e.g., Rad53 in *Saccharomyces cerevisiae*) that lead to specific responses such as the maintenance of replication fork stability, inhibition of late replication origins, DNA repair modulation and cell cycle arrest [3]. The presence of a sister chromatid provides a unique opportunity to repair and rescue the forks by homologous recombination (HR), even though the molecular mechanisms by which HR repairs and/or tolerates replicative DNA damage remain unclear [4].

In eukaryotes DNA is packaged into a highly specialized and dynamic nucleoprotein structure called chromatin, which is actually the substrate for cell machineries that deal with DNA. The repetitive unit of chromatin, the nucleosome, is formed by ∼146 base pairs of DNA wrapped 1.65 times around an octamer of histones. Nucleosome assembly of the replicated DNA is conducted by histone chaperones and chromatin assembly factors that first deposit two heterodimers of histones H3 and H4 to form a core (H3/H4)_2_ tetramer to which an H2A/H2B dimer binds on each side [5]. This provides the substrate for a plethora of ATP-dependent remodeling and histone modifier complexes that will eventually set up the specific chromatin structures required for the regulation of each DNA metabolic process. Replication coupled (RC)-chromatin assembly occurs rapidly after the passage of the replication fork and involves physical interactions between components of the replisome with chromatin assembly and remodeling factors; e.g., the replication processivity factor PCNA interacts with the chromatin assembly factor CAF1 [6], [7], the PCNA loader RFC with the histone chaperone Asf1 [8] and the MCM helicase complex with Asf1 and the chromatin remodeling complex FACT [9]–[11]. These interactions may facilitate nucleosome assembly but also help disrupt chromatin ahead of the fork. Besides, these interactions have been proposed to coordinate the flow of histones ensuring the exact supply at the fork [10], a process that is also regulated at the level of DNA and histone synthesis during the cell cycle [12]–[14].

Newly synthesized histones H3 and H4 are acetylated before being deposited at the fork, and this modification is required for nucleosome assembly [15]–[19]. Histone H4 is acetylated at lysines 5 and 12 by the acetyltransferase Hat1, this acetylation pattern being highly conserved from yeast to humans [15], [20], [21]. Histone H3 is also acetylated at its amino terminal tail, though the pattern is more variable among organisms. In the budding yeast H3 is acetylated at lysines 9 and 27 by the acetyltransferases Rtt109 and Gcn5 [22]. Additionally, histone H3 and H4 are acetylated in their globular domains at positions K56 and K91 by Rtt109 and Hat1, respectively [19], [23]–[26]. A detailed molecular analysis in yeast has recently deciphered part of the mechanisms of H3/H4 deposition during DNA replication. Thus, Asf1 binds to newly synthesized H3/H4 dimers [27] and presents them for acetylation of H3K56 by Rtt109 [23], [24]. This histone modification enhances the binding affinity of H3 to the chromatin assembly factors CAF1 and Rtt106 and of CAF1 to PCNA, thus promoting histone deposition at the proximity of the fork [17]. This process is also facilitated by direct interactions between CAF1 with Asf1 and Rtt106 and Asf1 with Rtt109 [26],[28]–[30]. Similarly, lysine acetylation at the amino terminal tail of H3 by Gcn5 enhances histone binding to CAF1 and Rtt106 and promotes RC chromatin assembly [16], suggesting that lysine acetylation might be a general mechanism to regulate the interaction of histones with chromatin assembly factors. In addition to newly synthesized histones, cells recycle parental histones that result from the disassembly of the chromatin ahead of the replication fork, a process in which Asf1 is also involved [10].

A number of results have clearly shown over the last few years that defective chromatin assembly causes genetic instability. In plants and human cells, the absence of CAF1 causes inhibition of DNA synthesis, accumulation of DNA damage and activation of the S-phase checkpoint [31], [32]. In yeast the disruption of a Gcn5-containing complex causes an accumulation of recombinogenic DNA damage [16], while the absence of H3K56 acetylation in *asf1Δ*, *rtt109Δ* and *H3K56R* mutants increases the frequency of HR and gross chromosomal rearrangements (GCRs) [23], [24], [33], [34]. Similarly, defective chromatin assembly by partial depletion of histones causes replication defects and hyper-recombination [35], [36]. In addition to the accumulation of DNA damage, chromatin assembly mutants are usually sensitive to genotoxic agents that impair DNA replication; thus, acetylation of H3K56 and lysines at the amino terminal tails of H3 and H4 prevent DNA damage sensitivity by non-redundant mechanisms [17], [23]–[25], [27], [37], [38]. Similarly, a mutant lacking Cac1 – the largest subunit of CAF1 – and Rtt106 is defective in RC-chromatin assembly and replicative DNA damage repair/tolerance [17]. However, the mechanisms by which chromatin assembly prevents the accumulation of DNA damage and the sensitivity to replicative DNA damage remain unknown. This is in part due to the fact that many of the players functioning in RC-chromatin assembly do it as well in replication independent chromatin assembly processes like DNA repair and checkpoint recovery; e.g., Asf1 and CAF1 are required for chromatin assembly and checkpoint turning off upon DNA double-strand break (DSB) repair [39]–[41]. In addition, it is difficult to discern whether the role of a histone mark in the DNA damage response (DDR) is prior or subsequent to histone deposition and whether it has a coding or a structural role.

We have recently shown that defective chromatin assembly by partial depletion of H4 is rapidly followed by the collapse of replication forks, which are efficiently rescued via HR, suggesting that correct nucleosome deposition is required for replication fork stability [42]. This approach, however, needs to be validated for specific chromatin assembly mutants. Here we have dissected the H3K56ac-dependent CAF1 and Rtt106 chromatin assembly pathways in terms of HR, checkpoint activation, replication fork stability and response to different genotoxic agents. Our results indicate that defective nucleosome assembly by impairment of H3K56ac-dependent CAF1 and Rtt106 pathways increases HR by affecting the integrity of advancing, but not stalled, replication forks. In contrast, H3K56ac is required after replicative DNA damage for CAF1/Rtt106-independent DNA repair/tolerance mechanisms that are likely to occur after its incorporation into chromatin.

## Results

### Defective replication-coupled H3/H4 deposition causes recombinogenic DNA damage and checkpoint activation

**Figure 1:**
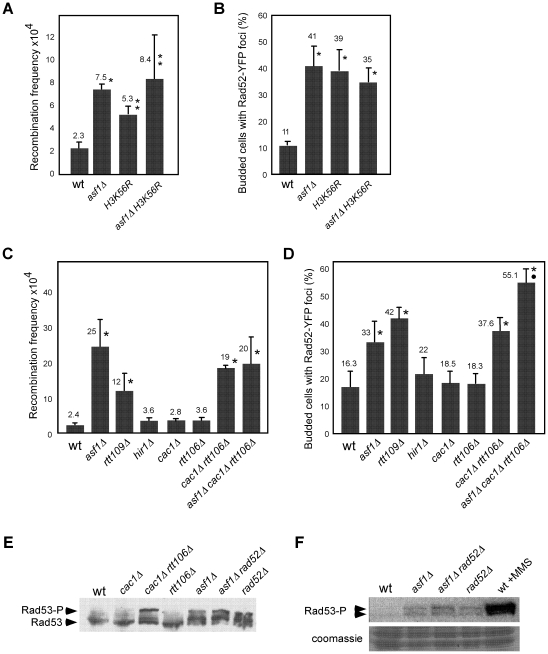
Defective replication-coupled chromatin assembly causes accumulation of recombinogenic DNA damage and checkpoint activation. Effect of *asf1Δ*, *H3K56R*, *asf1Δ H3K56R*, *rtt109Δ*, *hir1Δ*, *cac1Δ*, *rtt106*, *cac1Δ rtt106Δ* and *asf1Δ cac1Δ rtt106Δ* on the frequency of genetic recombination between inverted repeats (A, C) and budded cells with Rad52-YFP foci (B, D). Asterisks and circles indicate statistically significant differences compared to wild type and mutants *asf1Δ* and *cac1Δ rtt106Δ*, respectively, according to an Anova one-way (Tukey) test, where one asterisk/circle represents a *P*-value<0.001 and two represents <0.05. Note that strains in panels (A, B) and (C, D) have different genetic backgrounds (MSY421 and BY4701, respectively). For the frequency of genetic recombination the average and standard deviation of 3–16 fluctuation tests performed with 3–8 independent transformants of each strain are shown. For the percentage of budded cells with Rad52-YFP foci 600–900 cells for each strain were analyzed, and the average and standard deviation of 6–9 independent measures are shown. Rad53 phosphorylation in the indicated strains under unperturbed conditions by western blot (E) and *in situ* kinase assay (F). The wild-type strain treated with 0.033% MMS for 2 h was used as a control of checkpoint activation.

The histone chaperone Asf1 interacts with the histone acetyltransferase Rtt109, and both proteins are required for acetylation at lysine 56 of newly synthesized histone H3 [23], [24], [26], [43]. Consistent with a role for this histone modification in preventing DNA damage accumulation, the absence of H3K56 acetylation in *asf1Δ*, *rtt109Δ* and *H3K56R* mutants increases the frequency of genetic recombination and budded cells with foci of the recombination protein Rad52 fused to the yellow-fluorescence protein (Rad52-YFP) (Figure 1; [23], [24], [34]). As previously shown for *rtt109Δ* [24], we confirmed that the increase in recombination mediated by *asf1Δ* was due to its incapability acetylating H3 on lysine 56, as the frequency of genetic recombination and Rad52-YFP foci in *asf1Δ H3K56R* was as in the single mutants (Figure 1A and 1B).

Histone H3K56 acetylation marks nucleosomes incorporated into chromatin via both RC and replication independent mechanisms [44], [45]. Thus, we first assessed whether the observed increase in recombination was linked to defects in replication-independent chromatin assembly. In this regard, Asf1 interacts with the HIR complex (formed by Hir1, Hir2 and Hir3 in yeast) [46] with which promotes replication-independent chromatin assembly [47]. We analyzed recombination in the absence of Hir1 since this subunit is required for the integrity and histone deposition activity of Asf1/HIR [47]. As shown in Figure 1C and 1D, disruption of the HIR complex in *hir1Δ* did not affect recombination.

Acetylation of H3K56 is also involved in RC-nucleosome assembly. It promotes both the transfer of H3/H4 to the chromatin assembly factors CAF1 and Rtt106 and the binding of CAF1 to PCNA [17]. Consequently, hyper-recombination in *asf1Δ*, *rtt109Δ* and *H3K56R* could be associated with defective histone deposition but also with a loss of structural and/or coding information because of the absence of H3K56ac at chromatin. To distinguish between these possibilities we analyzed the role of CAF1 and Rtt106 in preventing the accumulation of recombinogenic DNA damage; CAF1 and Rtt106 have redundant chromatin assembly functions as shown by the fact that *cac1Δ rtt106Δ*, but not *cac1Δ* and *rtt106Δ*, is defective in histone deposition [17]. Besides, the levels of H3K56ac are not affected and its deposition at chromatin is delayed but not prevented in *cac1Δ rtt106Δ* [17]. While the single mutants *cac1Δ* and *rtt106Δ* were not affected in HR, the double mutant *cac1Δ rtt106Δ* increased the frequency both of genetic recombination and budded cells with Rad52-YFP foci as compared to the wild type (Figure 1C and 1D), indicating that CAF1- and Rtt106-dependent chromatin assembly pathways prevent the accumulation of recombinogenic DNA damage. Besides, the triple mutant *asf1Δ cac1Δ rtt106Δ* displayed the same frequency of genetic recombination as *asf1Δ* and *cac1Δ rtt106Δ*, suggesting that H3K56ac avoids hyper-recombination through its function in CAF1/Rtt106-dependent chromatin assembly. Nevertheless, the triple mutants displayed a slight but significantly higher frequency of cells with Rad52 foci than *asf1Δ* and *cac1Δ rtt106Δ*, suggesting the existence of additional, non-overlapping functions of H3K56ac and CAF1/Rtt106 in preventing the accumulation of DNA damage.

Another feature of *asf1Δ*, *rtt109Δ* and *H3K56R* is the activation of the DNA damage checkpoint in the absence of DNA damaging agents as determined by partial phosphorylation of Rad53 [23], [48], [49]; as shown in Figure 1E, only the simultaneous absence of CAF1 and Rtt106 led to the activation of Rad53. Therefore, our results indicate that defective RC-nucleosome assembly causes accumulation of recombinogenic DNA damage and checkpoint activation. However, and strikingly, the absence of Rad52 did not increase the amount of phosphorylated Rad53 in *asf1Δ* as determined by western blot and *in situ* kinase assays (Figure 1E and 1F), suggesting that accumulation of recombinogenic DNA damage and checkpoint activation are not genetically linked.

### Chromatin assembly prevents the loss of replication intermediates

**Figure 2:**
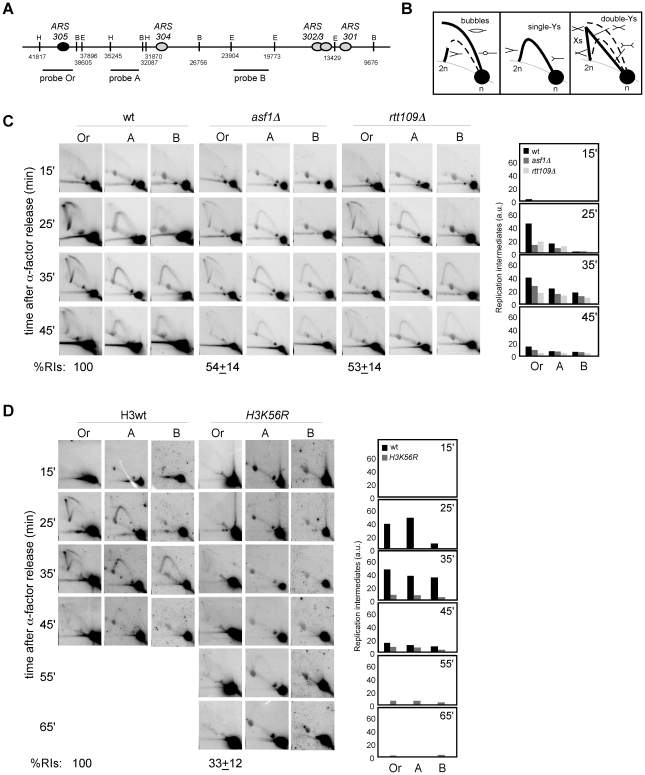
Histone H3K56 acetylation is required for preventing the loss of replication forks. (A) Schematic representation of the telomere-proximal region replicated from the early origin *ARS305* (black oval). The position of dormant origins (grey ovals) and restriction fragments analyzed by 2D-gel electrophoresis is shown. (B) Schematic representation of the migration pattern of the bubble-, single Y-, double Y- and X-shaped RIs by 2D-gel electrophoresis. (C, D) Analysis of RIs at the *ARS305* and two adjacent *Eco*RV-*Hin*dIII regions of cells synchronized in G1 and released into S phase. A representative kinetics with its quantification is shown. Quantification of the RIs was normalized to the total amount of DNA, including linear monomers (n), to the size of the restriction fragment, and to the percentage of cells synchronized in G1. The percentage of RIs at the *ARS305* during the kinetics was calculated as the sum of bubbles, Ys and Xs at region Or of all time points combined, taking the total amount of wild-type RIs as 100. The average and standard deviation of 5 (*asf1Δ*) and 3 (*rtt109Δ* and *H3K56R*) independent experiments are shown.

Histone deposition and DNA synthesis are tightly connected during DNA replication. We therefore hypothesized that defective nucleosome assembly in *asf1Δ*, *rtt109Δ*, *H3K56R* and *cac1Δ rtt106Δ* mutants might affect replication fork integrity, which in turn would generate genetic instability. To address this possibility we followed the fate of replication intermediates (RIs) in wild type and mutants by 2D-gel electrophoresis. For this, cells were synchronized in G1 with α-factor and released into S phase, and DNA samples were analyzed at different times to follow the progression of replication forks from the early replication origin *ARS305* (Figure 2A). Replication initiation and early elongation can be followed with probe Or by the formation of a bubble arc that reverts to a single Y-arc of large Y-shaped molecules when forks cross the nearest restriction site (Figure 2B, left panel), while replication fork progression along adjacent restriction fragments can be followed with specific probes by the accumulation of a complete arc of single Y-shaped molecules (Figure 2B, central panel). Finally, converging forks and Holliday junction (HJ)-like structures can be detected by the accumulation of double Y- and X-shaped molecules, respectively (Figure 2B, right panel).

The amount of RIs at the origin during the kinetics (i.e., the sum of bubbles, Ys and Xs at region Or of all time points combined), taking the total amount of wild-type RIs as 100, was reduced to ∼50% in *asf1Δ* and *rtt109Δ* (Figure 2C). In agreement with this defect being mediated by the lack of acetylation at H3K56 in *asf1Δ* and *rtt109Δ*, the total amount of RIs in a *H3K56R* mutant was 33% (Figure 2D). An increased drop in RIs was noticed in *H3K56R* as compared to *asf1Δ* and *rtt109Δ* (Figure 2C and 2D), which might be due to either an additional effect by reduced levels of histones – strains in Figure 2D have one instead of two H3/H4 genes – or the specific change to arginine. Therefore, the absence of H3K56 acetylation causes a loss of RIs. It should be noted that this reduction was also observed at adjacent DNA fragments, even though the effect became less evident at fragment B because of the loss of synchrony in the peak of RIs as the forks move away from the origin.

**Figure 3:**
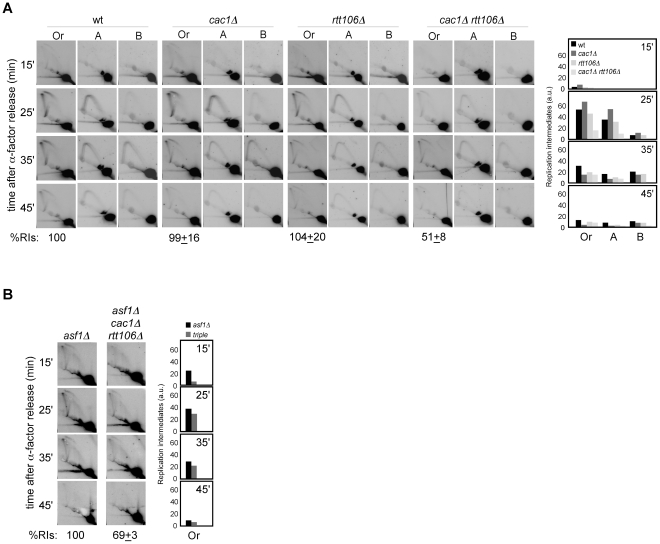
Defective CAF1/Rtt106-dependent chromatin assembly causes a loss of replication forks. (A, B) Analysis of RIs at the *ARS305* and two adjacent *Eco*RV-*Hin*dIII regions of cells synchronized in G1 and released into S phase. See Legend Figure 2 for details. The average and standard deviation of 3 independent experiments are shown.

Next, we decided to address whether the loss of RIs in mutants defective in H3K56 acetylation was due to defective chromatin assembly as previously shown for recombination and checkpoint activation. For this, the amount of replication forks from *cac1Δ*, *rtt106Δ* and *cac1Δ rtt106Δ* mutants synchronized in G1 and released into S phase was analyzed. As shown in Figure 3A, whereas the single mutants *cac1Δ* and *rtt106Δ* accumulated wild-type levels of RIs, the double mutant *cac1Δ rtt106Δ* displayed a ∼50% reduction in the amount of RIs at the origin, indicating that CAF1- and Rtt106-mediated chromatin assembly pathways have redundant roles in preventing the loss of replication forks. Besides, the levels of RIs in *cac1Δ rtt106Δ* were the same as in *asf1Δ* and *rtt109Δ* (∼50%), suggesting that the major role of H3K56 acetylation in replication fork stability is through its function in chromatin assembly. Consistently, the reduction in RIs in the triple mutant *asf1Δ cac1Δ rtt106Δ* was neither synergistic nor additive as compared to *asf1Δ* (69±3%; Figure 3B), though this drop opens the possibility that H3K56ac and CAF1/Rtt106 have also additional, non-overlapping functions in preventing the loss of RIs. Finally, we observed that the total amount of RIs at the replication origin *ARS315* was also significantly reduced in *asf1Δ* and *cac1Δ rtt106Δ* as compared to wild type (∼64 and ∼44%; Figure S1), indicating that the loss of RIs was not restricted to *ARS305*.

### Loss of RIs in chromatin assembly mutants is not associated with defects in *ARS305* initiation

In order to determine why defective chromatin assembly causes a loss of RIs, we first assessed the possibility that forks break during DNA extraction. Contrary to this, the loss of RIs in *asf1Δ* determined by collecting and digesting the DNA in agarose plugs to preserve its integrity was similar to that obtained with standard DNA extraction protocols (Figure S2).

**Figure 4:**
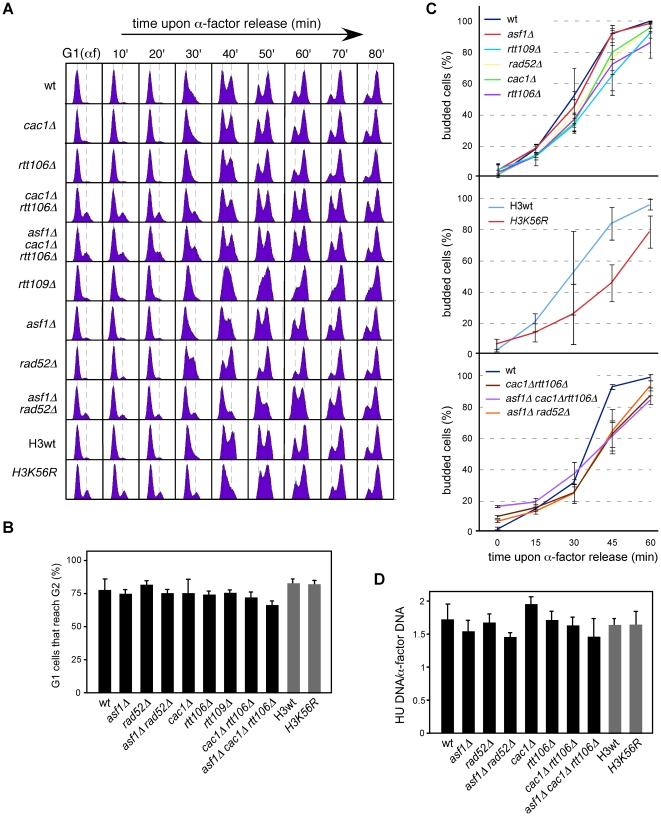
Chromatin assembly mutants are not affected in *ARS305* replication firing. (A) Cell cycle progression by DNA content analysis of cells synchronized in G1 and released into S phase. (B) Percentage of G1 synchronized cells that reach G2/M. This value was obtained by FACS analysis of cells synchronized in G1 and released into S phase in the presence of NCD until the number of cells in G2/M did not change. It was calculated as (%G2_f_-%G2_i_)/%G1_i_. The average and standard deviation of 3 independent experiments is shown. Statistically significant differences were not obtained according to an Anova one-way (Tukey) test. (C) Cell cycle progression by budding analysis of cells synchronized in G1 and released into S phase in the absence (top and middle) or presence (bottom) of NCD. The presence of NCD prevented G2/M cells at time cero from re-entering a new cell cycle thus allowing budding analysis in mutants in which α-factor synchronization led to less than 90% cells in G1. The average and standard deviation of 3 independent experiments are shown. Statistically significant differences compared to wild type (*P*-value<0.05) were obtained only in *cac1Δ rtt106Δ*, *asf1Δ cac1Δ rtt106Δ*, *asf1Δ rad52Δ* and *H3K56R* at times 45 and 60 minutes, according to an Anova one-way (Tukey) test. (D) Efficiency of *ARS305* replication firing determined as the amount of DNA at the origin in cells arrested in S phase with HU relative to cells arrested in G1 with α-factor. The average and standard deviation of 3 independent measures are shown. Statistically significant differences were not obtained according to an Anova one-way (Tukey) test.

Alternatively, this loss of RIs might be due to differences in replication initiation, either in the efficiency or in the synchrony of the firing. As a first approach to assess this possibility we analyzed cell cycle progression in chromatin assembly mutants. FACS and budding analyses showed that most G1 cells reached G2/M in all mutants (Figure 4A, 4B and 4C). Besides, neither *asf1Δ* nor *rtt109Δ* displayed a significant delay in completing S phase compared to the wild type (Figure 4A and 4C), suggesting that the loss of RIs in these mutants is not due to defects in replication initiation; in contrast, *H3K56R* was clearly retarded as compared with its wild type. Also, while *cac1Δ* and *rtt106Δ* were not affected, *cac1Δ rtt106Δ* mutants displayed a slight but significant delay (Figure 4A and 4C) that might influence the amount of RIs. However, the reduction in RIs in the triple mutant *asf1Δ cac1Δ rtt106Δ* was neither synergistic nor additive as compared to *asf1Δ* (Figure 3B), which is not affected in cell cycle progression. Therefore, the delay in the progression through S phase seems not to be the main cause for the loss of RIs in *cac1Δ rtt106Δ*, even though the 30% drop in the triple *asf1Δ cac1Δ rtt106Δ* versus the single *asf1Δ* mutant leaves open the possibility that a fraction of the drop in RIs reflects some defects in replication initiation.

Since FACS and budding analyses estimate whole genome duplication, we cannot rule out the possibility that cells progress normally through S phase but having problems in the firing of some specific origins that could be compensated with altered programs of initiation and/or elongation. Likewise, a slow advance through S phase does not necessarily reflect a defect at a specific replication origin. Therefore, we first asked whether the loss of RIs was a consequence of inefficient *ARS305* firing. In this regard, a defect in replication initiation would lead to a complete single Y-arc indicative of passive replication of the *ARS305* fragment by forks coming from a neighbor origin. Even though the shape of the single Y-arc in the mutants was as in the wild type (Figure 2 and Figure 3), we cannot discard that the region were replicated later either from *ARS305* or from a fork originated elsewhere. Therefore, we decided to determine the efficiency of replication initiation of the origin *ARS305*. Previous works have shown that *asf1Δ*, *rtt109Δ* and *H3K56R* are proficient in the activation of this origin [8], [50]. We studied replication initiation in our strains with a similar approach [42]; cells arrested in G1 with α-factor were released into S phase in the presence of hydroxyurea (HU) for 50 minutes, which causes the stalling of the forks in the proximity of the origin by depletion of available dNTPs. RT-PCR quantification of the total amount of DNA at the origin relative to an unreplicated fragment both in G1 and HU-arrested cells showed no significant defects in the firing of *ARS305* in any of the mutants tested (Figure 4D).

Next, we asked whether the loss of RIs was due to differences in the synchrony of the firing of replication from *ARS305*. Contrary to this possibility, chromatin assembly mutants displayed the same kinetics of RI accumulation as the wild type, with a peak for the *ARS305* region at 20–30 minutes upon G1 release (Figure 2 and Figure 3). This was not the case for *H3K56R*, in which the slow accumulation of RIs might explain its difference with *asf1Δ* and *rtt109Δ* (Figure 2D). Importantly, chromatin assembly mutants displayed a similar drop in RIs when released into S phase for 1 and 2 hours – what ensures that most cells have fired *ARS305* (Figure 4D) – in the presence of HU (see below), which stalls forks close to the origin and thereby minimizes putative differences in synchrony. Consequently, the loss of RIs in chromatin assembly mutants is not associated with defective replication initiation and therefore may reflect a loss of integrity of the replication forks as they move away from the origin.

### Homologous recombination is required for the rescue of collapsed replication forks in *asf1Δ*

**Figure 5:**
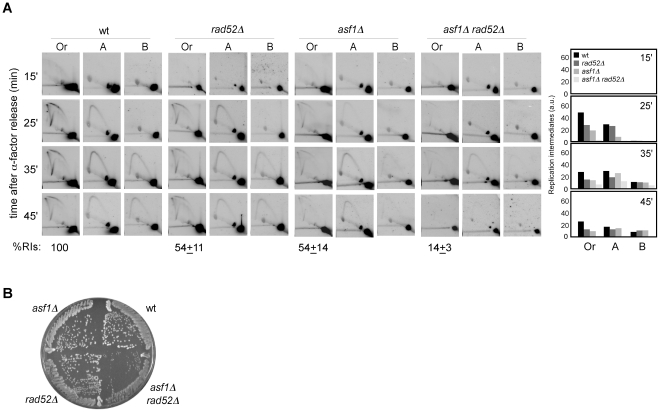
Homologous recombination is required for replication fork rescue in *asf1Δ*. (A) Analysis of RIs at the *ARS305* and two adjacent *Eco*RV-*Hin*dIII regions of cells synchronized in G1 and released into S phase. See Legend Figure 2 for details. The average and standard deviation of 3 independent experiments are shown. (B) Effect of *asf1Δ*, *rad52Δ* and *asf1Δ rad52Δ* on cell growth.

We have shown that chromatin assembly mutants display both a loss of RIs and an increase in recombination. Indeed, the stronger is the loss of RIs the higher is the percentage of cells with recombination foci. This correlation led us to hypothesize that the increase in recombination might result from the repair of collapsed replication forks. To address this possibility, we analyzed the role of Rad52, essential for DNA repair by HR [51], in the replication of cells lacking Asf1. As shown in Figure 5A, the amount of RIs dropped from about 54% in *asf1Δ* and *rad52Δ* to 14% in *asf1Δ rad52Δ*, being this drop not associated with defects in the kinetics of RI accumulation or in the firing of *ARS305* (Figure 4D). This synergistic reduction of RIs in *asf1Δ rad52Δ* suggests that HR participates in the rescue of collapsed forks from *ARS305* in *asf1Δ*. Consistently, *asf1Δ rad52Δ* cells displayed a delay in completing S phase (Figure 4A and 4C). These results provide an explanation for the accumulation of recombinogenic DNA damage in chromatin assembly mutants and the slow growth of *asf1Δ rad52Δ* cells (Figure 5B; [49]).

### H3K56ac/CAF1/Rtt106-dependent chromatin assembly is not required for the stability and restart of stalled replication forks

**Figure 6:**
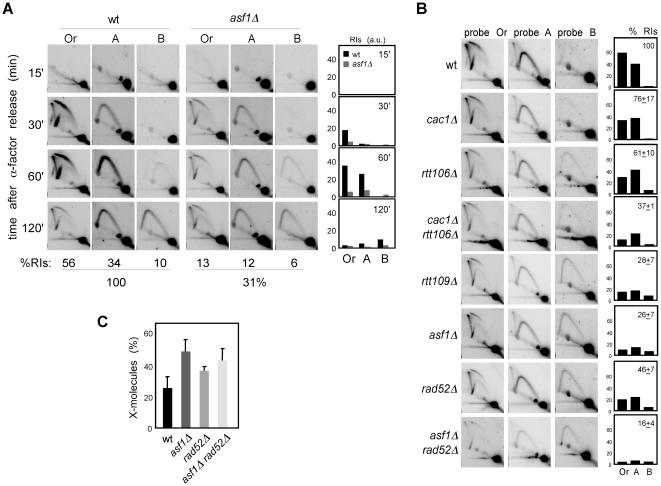
Chromatin assembly is not required for the stability of stalled replication forks. (A) Analysis of stalled RIs at the *ARS305* and two adjacent *Eco*RV-*Hin*dIII regions of cells synchronized in G1 and released into the S phase in the presence of 0.2 M HU for different times. The percentage of RIs over the whole region during the kinetics was calculated as the sum of bubbles, Ys and Xs of all time points combined, taking the total amount of wild-type RIs as 100. (B) Analysis of stalled RIs at the *ARS305* and two adjacent *Eco*RV-*Hin*dIII regions of cells synchronized in G1 and released into the S phase in the presence of 0.2 M HU for 1 hour. A representative kinetics with its quantification is shown. The percentage of RIs over the whole region was calculated as the sum of bubbles, Ys and Xs in the three fragments (Or, A and B), taking the total amount of wild-type RIs as 100. The average and standard deviation of 7 (*asf1Δ*) and 3 (rest) independent experiments are shown. (C) Amount of X-shaped molecules relative to total RIs (bubbles, Ys and Xs) at the *Eco*RV-*Hin*dIII *ARS305* fragment from cells synchronized in G1 and released into the S-phase in the presence of 0.2 M HU for 30 and 60 minutes. The average and standard deviation of 10 (*asf1Δ*), 6 (*rad52Δ*) and 7 (*asf1Δ rad52Δ*) values are shown. Only increases in *asf1Δ* (*P*-value<0.001), *asf1Δ rad52Δ* (*P*-value<0.001) and *rad52Δ* (*P*-value<0.01) relative to wild type, and in *asf1Δ* relative to *rad52Δ* (*P*-value<0.005) are statistically significant, according to an Anova one-way (Tukey) test.

Defective H3K56 acetylation in *asf1Δ*, *rtt109Δ* and *H3K56R* causes a reduction in the amount of ChIP-detected replisome components in the presence of HU that has been thought to be responsible for their high sensitivity to drugs that stall replication forks [8], . Those experiments, however, do not provide information about the integrity of DNA at the fork and cause of the collapse, which could be a defect in chromatin assembly but also the absence of H3K56 acetylation at chromatin. Besides, our previous results suggest a role for this modification in keeping the stability of unperturbed replication forks, leaving its role unresolved on stalled replication forks. Therefore, we followed the fate of RIs in cells synchronized in G1 and released into the S phase in the presence of HU, which leads to the stalling of the wild-type forks at the proximity of the origin with a peak of RIs at 60 minutes upon α-factor release (Figure 6A; [42], [52]). A similar kinetics of replication fork stalling was observed in *asf1Δ* (Figure 6A), indicating that synchrony was not affected; however, and consistent with previous ChIP analysis [8], [26], [50], the total amount of stalled RIs over the whole region (i.e., the sum of bubbles, Ys and Xs of all fragments, either of all time points combined (Figure 6A) or at 1 hour (Figure 6B)), taking the total amount of wild-type RIs as 100, dropped to ∼30% in *asf1Δ* and *rtt109Δ* (Figure 6A and 6B) and this reduction was not due to a distinctive distribution of the stalled forks along the DNA (Figure S3). Also, a similar drop in RIs was observed in *cac1Δ rtt106Δ* (Figure 6B), indicating that proper chromatin assembly is required to prevent the loss of RIs in the presence of HU. Therefore, HU further decreases the amount of RIs in chromatin assembly mutants from approximately 50 to 30%.

In principle, this enhanced loss of RIs in the presence of HU might be linked to a role for chromatin assembly in keeping the stability of both advancing and stalled replication forks, but also to a defect in resuming DNA replication upon HR-dependent fork rescue as a consequence of the HU-induced depletion of available dNTPs. In this case, however, the HU would not have any additional effect on replication fork stability in the absence of Rad52. As previously shown [42], the amount of RIs in *rad52Δ* was not affected by HU (∼50%; Figure 5A and Figure 6B), indicating that Rad52 is not required for the stability of stalled replication forks but likely for the rescue of damaged replication forks. Importantly, the amount of RIs in *asf1Δ rad52Δ* was not affected by the presence of HU (∼15%; Figure 5A and Figure 6B), suggesting that Asf1, and by extension H3K56 acetylation, has a minor role in the stability of stalled replication forks. In addition, and consistent with the idea that HU partially prevents the restart of replication forks, *asf1Δ* cells released into S phase in the presence of HU displayed a 2-fold increase in X-shaped molecules (Figure 6C). Unfortunately, the slight accumulation of X-shaped molecules in *rad52Δ* leaves an insufficient margin to determine the Rad52 dependency of the X-shaped molecules accumulated in *asf1Δ*.

**Figure 7:**
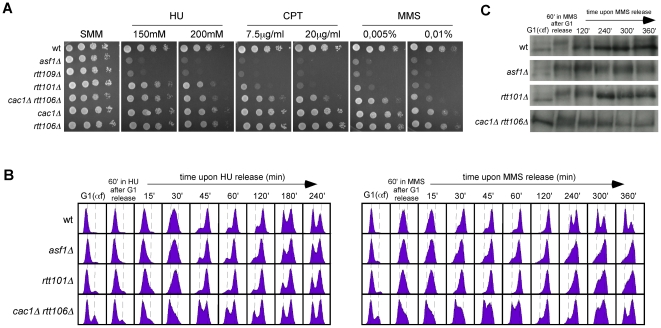
Roles of H3K56 acetylation and CAF1/Rtt106 on response to replication inhibition and replicative DNA damage. (A) DNA damage sensitivity to genotoxic agents as determined by ten-fold serial dilutions from the same number of mid-log phase cells onto medium containing drugs at the indicated concentrations. (B) Cell-cycle progression by FACS analysis of cells synchronized in G1 and released into the S-phase in the presence of 0.2 M HU (left) or 0.033% MMS (right) for 1 hour, and then released into fresh media for the indicated times. (C) Kinetics of checkpoint activation and deactivation upon replicative DNA damage as determined by western blot against phosphorylated Rad53 from selected samples in (B).

These results argue against a defect in the stability of stalled replication forks as a causative factor of the high sensitivity of *asf1Δ*, *rtt109Δ* and *H3K56R* to HU. Accordingly, the double mutant *cac1Δ rtt106Δ* was not sensitive to HU (Figure 7A), despite this strain displaying a similar loss of RIs as *asf1Δ* and *rtt109Δ*. In agreement with the growth assay, *cac1Δ rtt106Δ* was not required for stalled forks restart as determined by treating G1 released cells with 200 mM HU for 1 hour and checking their ability to resume DNA replication by FACS analysis (Figure 7B) (note that *cac1Δ rtt106Δ* displayed a similar delay during the S phase in the absence of HU (Figure 4A)). Strikingly, *asf1Δ* cells also resumed DNA replication after 1 hour in 200 mM HU and progressed to the following cell cycle without previous arrest (Figure 7B); consistently, *asf1Δ* cells did not display defects in checkpoint recovery and were viable (data not shown; [53], [54]). In summary, H3K56ac/CAF1/Rtt106-mediated chromatin assembly has no role in the stability and restart of forks stalled by HU, and therefore the loss of RIs observed in HU has to be of advancing replication forks.

### A CAF1/Rtt106-independent function of H3K56 acetylation promotes DNA repair and/or checkpoint recovery of damaged replication forks

Our previous results indicate that the role of H3K56ac in preventing sensitivity to chronic treatment with HU is independent of CAF1/Rtt106, suggesting that is a function separate from chromatin assembly and likely subsequent to its deposition at chromatin. A global epistatic analysis of pairs of gene deletions revealed a connection between Asf1 and Rtt109 with the Rtt101 ubiquitin ligase complex [53], which appear to promote fork progression through damaged DNA by HR [55]–[57]. However, as previously shown and in contrast to *asf1Δ* and *rtt109Δ*, *rtt101Δ* was not sensitive to HU (Figure 7A; [56], [57]).

H3K56ac, and by extension Asf1 and Rtt109, are also required for growth in the presence of drugs that impair the advance of the replication forks by DNA damage, such as the topoisomerase I inhibitor camptothecin (CPT) or the DNA alkylating agent methyl methane sulfonate (MMS) (Figure 7A; [23]–[25], [27], [39]). Again, these sensitivities could be associated with the role of H3K56ac in chromatin assembly. A comparative analysis showed that although the double mutant *cac1Δ rtt106Δ* was sensitive to both drugs, in particular to high concentrations, this sensitivity was much milder than that displayed by *asf1Δ* and *rtt109Δ* (Figure 7A, see CPT at 7.5 µg/ml and MMS at 0.005%), suggesting that the main role of H3K56ac in response to CPT and MMS is also independent of CAF1/Rtt106 and subsequent to its deposition into chromatin. The ubiquitin ligase complex Rtt101 has been shown to be required for MMS- and CPT-induced HR [55] and for checkpoint recovery (Figure 7C; [53], [55], [57]). Our comparative analysis showed that *rtt101Δ* was not as sensitive to MMS and CPT as *asf1Δ* and *rtt109Δ* (Figure 7A); thus, these results suggest that H3K56ac promotes fork progression through damaged DNA via Rtt101-mediated HR and, to a lesser extent, CAF1/Rtt106-mediated chromatin assembly.

To further understand the role of the CAF1/Rtt106 chromatin assembly pathway on MMS and CPT resistance, we analyzed the ability of *cac1Δ rtt106Δ* to resume DNA replication upon the treatment of G1 released cells with a high concentration (0.033%) of MMS. *cac1Δ rtt106Δ* cells resumed and completed DNA replication but remained partially arrested in mitosis (Figure 7B) as a consequence of a delay in checkpoint deactivation (Figure 7C), being these phenotypes much stronger in *asf1Δ* and *rtt101Δ* in agreement with the sensitivity assay.

## Discussion

### H3K56ac-, CAF1-, and Rtt106-dependent chromatin assembly pathways prevent the accumulation of recombinogenic DNA damage by keeping the stability of advancing replication forks

H3K56 acetylation is a histone modification required for chromatin assembly. Notably, mutants defective in H3K56 acetylation (*asf1Δ*, *rtt109Δ* and *H3K56R*) accumulate recombinogenic DNA damage as determined by genetic recombination, cells with Rad52 foci and molecular analysis of sister-chromatid exchange [23], [24], [34]. How H3K56 acetylation prevents DNA damage accumulation is not predictable, however, because its role in chromatin assembly is associated not only with replication but also with other processes that influence HR, such as transcription, silencing, DSB repair or DNA damage tolerance [58]. We first ruled out a role for replication-independent chromatin assembly as a disruption of the HIR/Asf1 complex in *hir1Δ* exhibited wild-type levels of recombination. Alternatively, and in agreement with a model in which spontaneous genetic instability stems from defective DNA damage repair/tolerance, hyper-recombination might result from defective repair/tolerance and channelling to HR of spontaneous DNA lesions. In this case, DNA damage induction with genotoxic agents to which these mutants are sensitive should further increase their levels of recombination. In contrast, Asf1, Rtt109 and the Rtt101 complex are required for HR induced by MMS and CPT [55]. Given that Asf1 and Rtt109 are not required for DSB-induced HR, both ectopic and sister-chromatid recombination [34], [49], [55], hyper-recombination in cells defective in H3K56 acetylation may be associated with the generation of DSBs. Accordingly, GCRs are mediated by the DSB-repair pathway of non-homologous end-joining and are prevented by HR in *asf1Δ* [33].

H3K56 acetylation enhances the binding affinity of H3 to CAF1 and Rtt106, two factors with redundant histone deposition functions during replication [17]. We show that only the RC-chromatin assembly defective *cac1Δ rtt106Δ*, but not the RC-chromatin assembly proficient *cac1Δ* and *rtt106Δ*, leads to recombinogenic DNA damage and checkpoint activation, and that the main role of H3K56ac in preventing hyper-recombination is mediated by CAF1 and Rtt106. Therefore, RC-chromatin assembly prevents the accumulation of recombinogenic DNA damage.

We show that chromatin assembly mutants display a loss of RIs that is not due to defects in replication initiation, and that there is a correlation between the loss of RIs and the increase in HR. Besides, the absence of Rad52, essential for HR, further increases the loss of RIs in *asf1Δ*. These results, together with the reported loss of replisome integrity in H3K56 acetylation mutants in the presence of HU [8], [26], [50] despite the fact that they are not affected in the stability and rescue of stalled replication forks (Figure 6 and Figure 7), strongly suggest that defective RC-chromatin assembly causes a loss of integrity of the advancing replication forks, and that HR participates in the rescue of these forks using the sister chromatid. Consistent with this, *asf1Δ* accumulates spontaneously sister-chromatid exchange products [34].

This loss of integrity may end up in the collapse of some of the forks, which can render unprotected DNA ends susceptible of being processed by HR [59]–[62] but that are difficult to be detected by 2D-gel analysis unless a homogeneous and stable population of intermediates accumulates. In particular, the detection of broken intermediates is not easy because the breakage of single Ys leads to linear molecules, while the breakage of bubbles leads to a mixture of asymmetric Ys that do not run at a defined arc. Additionally, defective chromatin assembly might generate DNA structures that are lost due to the running conditions required for the visualization of the RIs by 2D-gel analysis. Similarly, the reduction in the total amount of detectable RIs in chromatin assembly mutants in spite of the fact that they complete replication opens the possibility that the rescue of the collapsed forks and subsequent completion of DNA replication are not associated with the formation of a canonical replication fork [63] or reflects an asynchronous fork rescue along the DNA region. Finally, we cannot rule out that a fraction of the drop in the amount of RIs to be a consequence of problems in the initiation of replication of a subpopulation of cells as suggested by the analysis of cell cycle progression in *cac1Δ rtt106Δ* mutants.

Strikingly, defective chromatin assembly hardly affected (*asf1Δ*, *rtt109Δ*) or delayed just 10–20 minutes (*H3K56R*, *cac1Δ rtt106*, *asf1Δ cac1Δ rtt106Δ*) the time required for DNA duplication despite the loss of RIs. Replication fork rescue by HR cannot account for completion of DNA replication because *asf1Δ rad52Δ* cells are also capable of completing DNA duplication (Figure 4). Additional mechanisms may operate in the rescue of the collapsed replication forks; in this regard, it has recently been shown that *asf1Δ* accumulates ribosomal DNA repeats by a novel mechanism that is independent of HR but needs replication processivity functions known to be required for break-induced replication [64]. This work is consistent with our proposal that chromatin assembly mutants accumulate broken forks and that there may be mechanisms other than HR involved in the repair of these breaks. We have observed that the loss of RIs is not specific of forks coming from *ARS305* (Figure S1); however, we cannot rule out the possibility that not all chromatin regions display the same replication defects, that a proportion of the forks are functional but are lost during the 2D-gel analysis, and that chromatin assembly mutants counteract the instability of the replication forks by altering the program of replication initiation and/or increasing the rates of replication elongation. In this frame, it is possible that an “open” chromatin structure in these mutants favors alternative outputs of collapsed fork rescue and DNA replication as suggested above. Genome-wide analyses have to be conducted to address these possibilities.

Why are replication forks unstable under conditions of defective RC-chromatin assembly? These mutants are proficient in checkpoint activation (Figure 1 and Figure 7; [23], [34], [48], [49], [53], [65]), ruling out a defect in this mechanism of replication fork stability as responsible for the loss of RIs. In fact, the absence of checkpoint proteins in *asf1Δ* affects cell progression during the S phase [54], suggesting that chromatin assembly and replication checkpoints have non-redundant functions in replication fork stability. In principle, the loss of RIs and the increase in HR could be associated with defects in chromatin structure as a consequence of the lack of H3K56 acetylation at chromatin. This modification breaks a water-mediated histone-DNA interaction at the point of entry and exit of the nucleosomal DNA that modulates chromatin compaction [25], [66]–[68]. Also, this modification might recruit chromatin factors required for fork stability. We do not favor these possibilities in *cac1Δ rtt106Δ* because this mutant expresses acetylable H3K56, although its deposition at chromatin appears to be delayed and might generate regions behind the fork with reduced H3K56ac [17].

Alternatively, replication fork instability might result from defective chromatin disassembly and/or transfer of parental histones ahead of the fork. In this regard, Asf1, which is also a nucleosome disassembly factor [69], interacts with MCM to coordinate fork progression and parental histone supply ahead of the fork [10]. However, *asf1Δ* and *H3K56R* mutants share similar defects in replication fork stability and HR and the effect of *asf1Δ* is due to defective H3K56 acetylation as determined by epistatic analysis. Since this modification marks preferentially newly synthesized histones [25], our results point to defects in the pathway of newly synthesized histone deposition as the main cause of fork collapse and subsequent repair by HR.

DNA synthesis and histone deposition are physically and genetically connected to ensure the exact supply of histones at the fork [6]–[11]. Histone excess is toxic and cells are endowed with different mechanisms to get rid of non-incorporated histones [12]. The opposite situation, a reduction in the pool of available histones, is also deleterious and phenocopies the defects in fork stability and HR reported here with RC-chromatin assembly mutants [42]. The current study provides additional support to the idea that, under conditions of defective H3/H4 deposition during replication, DNA synthesis and nucleosome assembly could become uncoupled exposing DNA fragments behind the fork. This uncoupling might favor the formation of unstable secondary DNA structures, as it has been proposed to explain the high levels of DNA breakage and contractions at CAG/CTG tracts displayed by *asf1Δ* and *rtt109Δ* but not *rtt101Δ* [70]. Although these structures could be targeted by nucleases, we failed to find single nuclease mutants that alter the frequency of RI loss in *asf1Δ* (data not shown), a result that is not unexpected because of the redundancy of DNA nucleases in DNA damage repair [71], [72]. Finally, the loss of RIs and the increase in HR could be due to defective stability of stalled forks, as suggested by the observation that the replisome is unstable in the presence of HU in H3K56 acetylation mutants [8], [26], [50]. Here, we present some evidence indicating that only advancing, but not stalled forks, are affected in RC-chromatin assembly mutants. First, the total amount of RIs in chromatin assembly mutants defective in fork rescue by HR (*asf1Δ rad52Δ*) is not affected by the presence of HU. Second, RC-chromatin assembly mutants (*asf1Δ*, *rtt109Δ* and *cac1Δ rtt106Δ*) are proficient in stalled fork stability and restart upon an acute treatment with HU as determined by FACS analysis, checkpoint recovery and cell viability. Therefore, our results point to defects in the stability of advancing forks as the cause of the genetic instability in RC-nucleosome assembly mutants, further supporting the idea that defective histone deposition uncouples DNA synthesis and nucleosome assembly. Notably, *asf1Δ* cells treated with HU also exhibited an accumulation of Polα at the fork and an uncoupling of the MCM helicase [8]. We speculate that these alterations in the replisome structure might also occur in the absence of HU. Indeed, Asf1 interacts with MCM [10] and with RFC – which loads PCNA and in this way replaces Polα with Polε and Polδ – [8], and H3K56 acetylation regulates the function of the RFC [73]; it is thereby possible that the absence of Asf1 and/or H3K56ac could specifically alter the distribution of the polymerases and the MCM helicase at the fork.

### H3K56 acetylation protects against replicative DNA damage by DNA repair/tolerance mechanisms that are subsequent to the process of RC-nucleosome deposition

H3K56 acetylation – and by extent Asf1 and Rtt109 – is required for promoting resistance to replicative DNA damage [17], [23]–[25], [27]. Indeed, there is a correlation between the levels of H3K56 acetylation and the degree of DNA damage sensitivity to genotoxic agents [43]; consistently, *H3K56Q*, which mimics constitutive acetylation, suppresses *asf1Δ* sensitivity to HU and CPT [39], [43]. In contrast to H3K56 acetylation mutants, *cac1Δ rtt106Δ* is only sensitive to high concentrations of MMS and CPT and is not sensitive to chronic treatment with HU, suggesting that the function of H3K56ac in the replicative DNA damage response can be separated from its role in CAF1/Rtt106-mediated chromatin assembly. This points to a role subsequent to its deposition into chromatin. In agreement with this idea, it has recently been shown that a change of lysine 56 to glutamic acid in H3 generates a histone proficient in binding to CAF1 and Rtt106 but sensitive to replicative DNA damage [74]. An epistatic analysis has included Asf1, Rtt109 and the Rtt101 ubiquitin ligase complex into a functional group involved in DNA repair [53]. Rtt101 is recruited to chromatin in response to DNA damage in a process that requires Rtt109 [75], and Asf1, Rtt109 and Rtt101 promotes the repair of replicative DNA damage – but not DSBs – by SCE [34], [49], [55], suggesting that H3K56 acetylation might facilitate the repair of fork-associated DNA lesions other than DSBs by recruiting Rtt101, which in turn would promote HR. This model, however, would not be valid for HU sensitivity, which is Rtt101 independent, and may be related with sustained replication under conditions of low levels of dNTPs.

Besides, our comparative analysis shows that H3K56 acetylation mutants are slightly more sensitive to DNA damage than *rtt101Δ*, suggesting an additional function for this histone modification in response to replicative DNA damage. This role could be to open the chromatin and facilitate the access of repair proteins to DNA. Other possibility is that H3K56 acetylation promotes checkpoint deactivation via CAF1/Rtt106-chromatin assembly upon the repair of the replicative DNA damage, as previously demonstrated for DSB repair [39], [40]. This is supported by the fact that *cac1Δ rtt106Δ* becomes temporally arrested at mitosis by sustained phosphorylation of Rad53 upon DNA damage release, even though this defect might also be a consequence of an incomplete accumulation of H3K56ac behind the fork of the double mutant.

### Chromatin assembly and genome integrity in mammalian cells

Our results in yeast anticipate a similar role for chromatin assembly in the stability of advancing replication forks through the more demanding chromatin structure of mammalian genomes. It will thereby be well worth the effort to address replication fork integrity in human cells defective in RC-chromatin assembly, which are known to arrest in the S phase and accumulate DNA damage [13], [32], [36]. Finally, the results presented here reveal the process of RC-chromatin assembly as a potential target against cell proliferation in cancer therapy, as also suggested by a recent observation showing that human Asf1b is overexpressed in breast tumours [76].

## Materials and Methods

### Yeast strains, plasmids, and growth conditions

Yeast strains used in this study are listed in Table 1. They all are isogenic to BY4741, except for *H3K56R* mutants that are isogenic to MSY421. pRS316-SU [77] and pWJ1344 (kindly provided by R. Rothstein, Columbia University) are centromeric plasmids containing the SU inverted-repeat recombination system and *RAD52-YFP*, respectively. Yeast cells were grown in supplemented minimal medium (SMM), except for nocodazole (NCD) synchronization that were grown in YPD medium [78]. For G1 synchronization, cells were grown to mid-log-phase and α factor was added twice at 1.5 hours intervals at either 0.5 µg/ml (*asf1Δ rad52Δ*, *cac1Δ rtt106Δ* and *asf1Δ cac1Δ rtt106Δ*) or 0.25 µg/ml (rest of strains). Then, cells were washed three times and released into the S phase at different times in fresh medium with or without 0.2 M HU and 50 µg/ml pronase. Cell cycle progression was followed by DNA content analysis (data not shown). To prevent cells from re-entering a new cell cycle in Figure 4B and 4C (bottom), G1-synchronized cells were shifted to YPD with α factor for 1 hour and released into the S phase in fresh YPD medium with 50 µg/ml pronase and 15 µg/ml NCD.

**Table 1:** Strains.

*Strain*	*Relevant genotype*	*Source*
BY4741	*MATa his3Δ*1 *leu2Δ*0 *ura3Δ*0 *met15Δ*0	Euroscarf
BY4741b	*MATa his3Δ*1 *leu2Δ*0 *ura3Δ*0 *met15Δ*0 *bar1Δ::hyg*	Clemente-Ruiz 2009
BYrad52b-1D	*MATa his3Δ*1 *leu2Δ*0 *ura3Δ*0 *met15Δ*0 *bar1Δ::hyg rad52Δ::kan*	Clemente-Ruiz 2009
BYasf1b-7A	*MATa his3Δ*1 *leu2Δ*0 *ura3Δ*0 *bar1Δ::hyg asf1Δ::kan*	This work
BYasf1rad52b-11D	*MATa his3Δ*1 *leu2Δ*0 *ura3Δ*0 *met15Δ*0 *bar1Δ::hyg rad52Δ::kan asf1Δ::kan*	This work
BYcac1b-2B	*MATa his3Δ*1 *leu2Δ*0 *ura3Δ*0 *met15Δ*0 *bar1Δ::hyg cac1Δ::kan*	This work
BYrtt106b-21D	*MATa his3Δ*1 *leu2Δ*0 *ura3Δ*0 *bar1Δ::hyg rtt106Δ::kan*	This work
BYcac1rtt106b-3B	*MATa his3Δ*1 *leu2Δ*0 *ura3Δ*0 *met15Δ*0 *bar1Δ::hyg cac1Δ::kan rtt106Δ::kan*	This work
BYrtt109b-4D	*MATa his3Δ*1 *leu2Δ*0 *ura3Δ*0 *bar1Δ::hyg rtt109Δ::kan*	This work
BYacrb-2	*MATa his3Δ*1 *leu2Δ*0 *ura3Δ*0 *met15Δ*0 *bar1Δ::hyg cac1Δ::kan rtt106Δ::kan asf1Δ::nat*	This work
Y01376	*MATa his3Δ*1 *leu2Δ*0 *ura3Δ*0 *met15Δ*0 *rtt101Δ::kan*	Euroscarf
Y03034	*MATa his3Δ*1 *leu2Δ*0 *ura3Δ*0 *met15Δ*0 *hir1Δ::kan*	Euroscarf
MSY421	*MATa Δ(hht1-hhf1) Δ(hht2-hhf2) leu2-3*, *112*, *ura3-62*, *trp1*, *his3*, *pMS329 (HHT1-HHF1, URA3, CEN)*	Recht 2006
MSY421 asf1	*MATa Δ(hht1-hhf1) Δ(hht2-hhf2) leu2-3*, *112*, *ura3-62*, *trp1*, *his3*, *pMS329 (HHT1-HHF1, URA3, CEN) asf1Δ::kan*	Recht 2006
MSY421 K56R	*MATa Δ(hht1-hhf1) Δ(hht2-hhf2) leu2-3*, *112*, *ura3-62*, *trp1*, *his3*, *(hht2-K56R-HHF2, TRP1, CEN)*	Recht 2006
MSY421 K56R asf1	*MATa Δ(hht1-hhf1) Δ(hht2-hhf2) leu2-3*, *112*, *ura3-62*, *trp1*, *his3*, *(hht2-K56R-HHF2, TRP1, CEN) asf1Δ::kan*	Recht 2006
MSY421b	*MATa Δ(hht1-hhf1) Δ(hht2-hhf2) leu2-3*, *112*, *ura3-62*, *trp1*, *his3*, *pMS329 (HHT1-HHF1, URA3, CEN) bar1Δ::nat*	This work
MSY421b K56R	*MATa Δ(hht1-hhf1) Δ(hht2-hhf2) leu2-3*, *112*, *ura3-62*, *trp1*, *his3*, *(hht2-K56R-HHF2, TRP1, CEN) bar1Δ::nat*	This work

### Genetic recombination and DNA damage sensitivity

The frequency of Leu^+^ recombinants generated by recombination between inverted repeat sequences was determined in cells transformed with plasmid pRS316-SU by fluctuation tests as the median value of six independent colonies [77]. DNA damage sensitivity was determined by plating ten-fold serial dilutions from the same number of mid-log phase cells onto medium without or with genotoxic agents at the indicated concentrations.

### Analysis of Rad52-YFP foci

The proportion of budded cells with Rad52-YFP foci was performed as described previously [34]. Mid-log-phase cells transformed with pWJ1344 were visualized with Leica CTR6000 fluorescence microscope.

### Flow citometry and budding analyses

DNA content analysis was performed by fluorescence-activated cell sorting (FACS) as reported previously [35]. The percentage of budded cells was determined by counting 200 cells at each time point.

### Analysis of RIs

Each replication kinetic was conducted in parallel with the mutants and the wild type. Cell cultures were arrested with sodium azide (0.1% final concentration) and cooled down in ice. Total DNA was isolated either in agarose plugs or with the G2/CTAB protocol as previously reported [42], digested with restriction enzymes, resolved by neutral/neutral two-dimensional-gel electrophoresis as described [79], blotted to nylon membranes and analysed by sequential hybridization of the same membrane with different ^32^P-labelled probes (for probes along the *ARS305* region see [42]; probe for *ARS315* was PCR amplified with oligos AACAGCTTCTCTTGCCGTAG and TGTACTGAACCTACCGCTCC). All signals were quantified using a Fuji FLA5100 and ImageGauge as analysis program. Quantification of the RIs was normalized to the total amount of DNA, including linear monomers (n), to the size of the restriction fragment, and to the percentage of cells synchronized in G1; thus, the total amount of RIs at each specific region and time point was calculated as [ΣRIs/Σ(RIs+n×*g*)]×*f*, where *f* is the ratio between the size of the DNA fragment containing the origin and the size of the specific DNA fragment, and *g* is the proportion of cells in G1 after α-factor synchronization.

### Analysis of *ARS305* replication firing efficiency

Total DNA from mid-log phase cells synchronized in G1 and released into S phase in the presence of 0.2 M HU for 50 minutes was extracted and the amount of DNA at the origin *ARS305* and a non-replicated control region (located at ∼7 kb from the late replicating origin *ARS609*) determined by qPCR (*ARS305*: oligos CGCCCGACGCCGTAA and GAGCGGCCTGAAATACTGTCA; control region: oligos TACACCAGCCCGGATTTAAG and GACCAGTGGCTGAGTCACAA). The efficiency of replication initiation was calculated as the ratio between the amount of DNA in HU-arrested cells and the amount of DNA in G1-arrested cells at the origin normalized to the same ratio at the control DNA region.

### Western blot and *in situ* kinase assay

Yeast protein extracts were prepared from mid-log-phase cultures using the TCA protocol as described [35] and run on a 8% and 10% sodium dodecyl sulfate-polyacrilamyde gel for western blot and *in situ* kinase assay, respectively. Rad53 was detected either with rabbit polyclonal antibody JDI47 [80] (Figure 1E) or with goat polyclonal antibody (yC19) (Santa Cruz Biotechnology, INC) (Figure 7C). The autophosphorylation reaction was performed as described [81].

## Supporting Information

Figure S1 Defective chromatin assembly in *asf1Δ* and *cac1Δ rtt106Δ* causes a loss of RIs at *ARS315*. Analysis of RIs at the *Eco*RI fragment encompassing the *ARS315* origin of cells synchronized in G1 and released into S phase. A representative kinetics with its quantification, as well as the average and standard deviation of RIs at the *ARS315* during the kinetics of 3 independent experiments, are shown.
(TIF)

Figure S2 Analysis of RIs at the *ARS305* with DNA collected and restricted in agarose plugs. DNA from wild type and *asf1Δ* cells released into S phase upon G1 synchronization was extracted and restricted with *Eco*RV and *Hind*III in agarose plugs and analyzed by 2D-gel electrophoresis. Quantification of RIs, taken the total amount of wild-type RIs over the region as 100, is shown.
(TIF)

Figure S3 Analysis of stalled RIs at the *ARS305* and four adjacent regions of cells synchronized in G1 and released into the S phase in the presence of 0.2 M HU for 1 hour. Quantification of RIs, taken the total amount of wild-type RIs over the region as 100, is shown. A schematic representation of the telomere-proximal region replicated from the early origin *ARS305* with the restriction fragments analyzed by 2D-gel electrophoresis is also shown on top.
(TIF)
