# Supplementary figures and images for: Histone H3K56 Acetylation, CAF1, and Rtt106 Coordinate Nucleosome Assembly and Stability of Advancing Replication Forks
Source: PLoS Genet. 2011 Nov 10;7(11):e1002376. doi: 10.1371/journal.pgen.1002376 (PMC3213180; doi:10.1371/journal.pgen.1002376)

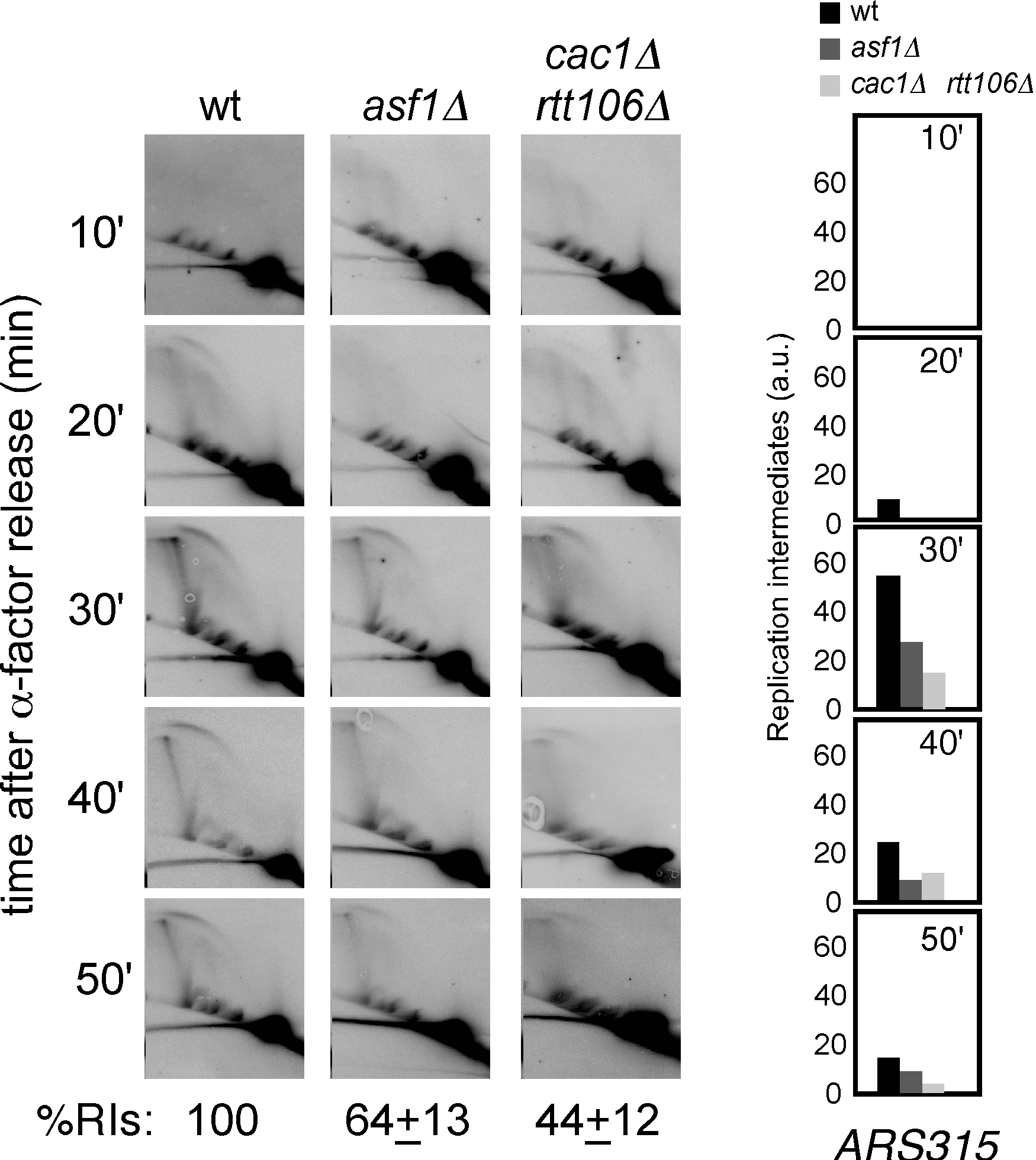

Supplement: Figure S1 — Defective chromatin assembly in asf1Δ and cac1Δ rtt106Δ causes a loss of RIs at ARS315. Analysis of RIs at the EcoRI fragment encompassing the ARS315 origin of cells synchronized in G1 and released into S phase. A representative kinetics with its quantification, as well as the average and standard deviation of RIs at the ARS315 during the kinetics of 3 independent experiments, are shown. (TIF) [file pgen.1002376.s001.tif]

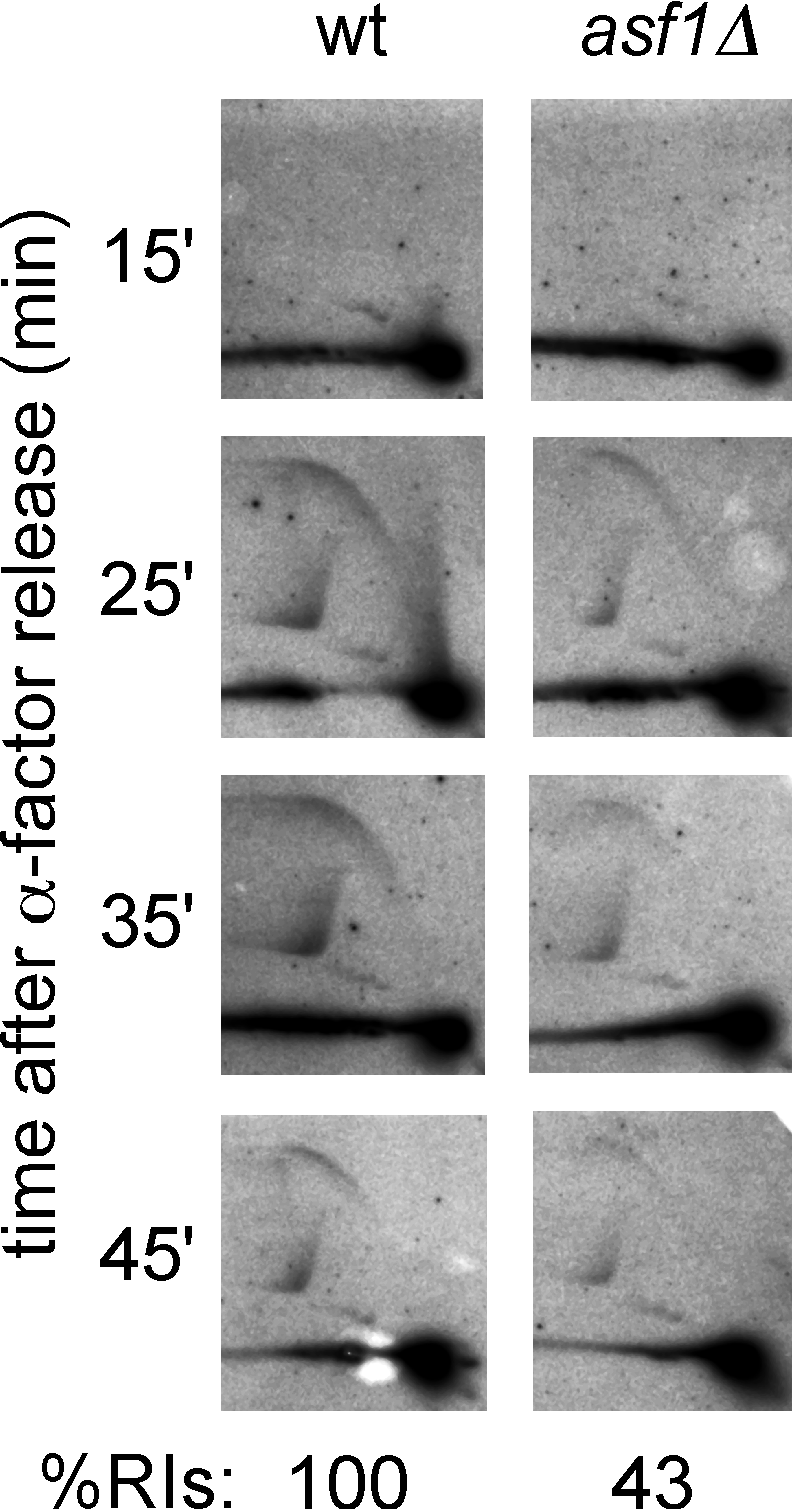

Supplement: Figure S2 — Analysis of RIs at the ARS305 with DNA collected and restricted in agarose plugs. DNA from wild type and asf1Δ cells released into S phase upon G1 synchronization was extracted and restricted with EcoRV and HindIII in agarose plugs and analyzed by 2D-gel electrophoresis. Quantification of RIs, taken the total amount of wild-type RIs over the region as 100, is shown. (TIF) [file pgen.1002376.s002.tif]

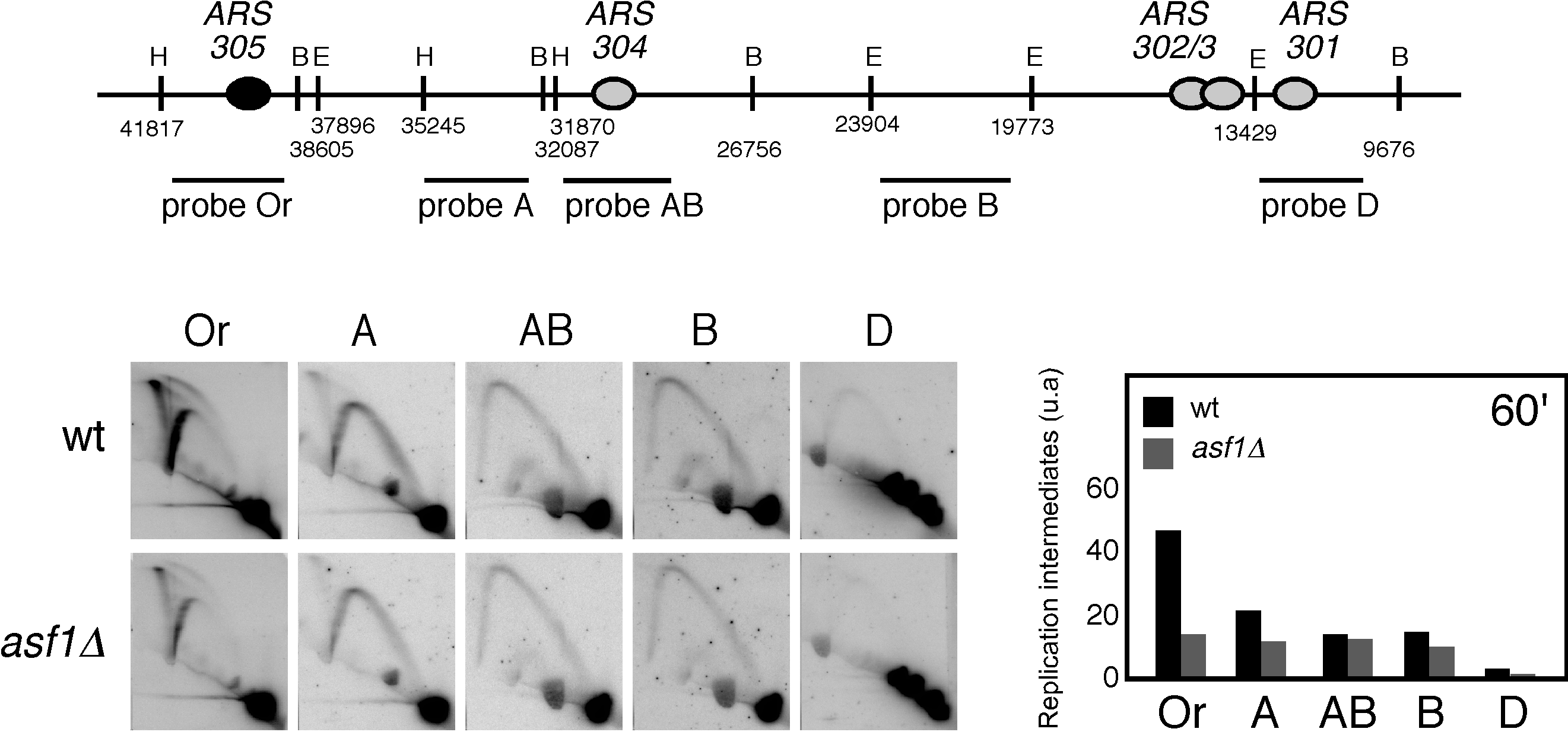

Supplement: Figure S3 — Analysis of stalled RIs at the ARS305 and four adjacent regions of cells synchronized in G1 and released into the S phase in the presence of 0.2 M HU for 1 hour. Quantification of RIs, taken the total amount of wild-type RIs over the region as 100, is shown. A schematic representation of the telomere-proximal region replicated from the early origin ARS305 with the restriction fragments analyzed by 2D-gel electrophoresis is also shown on top. (TIF) [file pgen.1002376.s003.tif]
